# Differences in Virulence Between *Legionella pneumophila* Isolates From Human and Non-human Sources Determined in *Galleria mellonella* Infection Model

**DOI:** 10.3389/fcimb.2018.00097

**Published:** 2018-04-04

**Authors:** Patrícia S. Sousa, Inês N. Silva, Leonilde M. Moreira, António Veríssimo, Joana Costa

**Affiliations:** ^1^Department of Life Sciences, University of Coimbra, Coimbra, Portugal; ^2^Institute for Bioengineering and Biosciences, Instituto Superior Técnico, University of Lisbon, Lisbon, Portugal; ^3^Department of Bioengineering, IST, University of Lisbon, Lisbon, Portugal; ^4^Centre for Functional Ecology - Science for People & the Planet, Department of Life Sciences, University of Coimbra, Coimbra, Portugal

**Keywords:** *Legionella pneumophila*, *Galleria mellonella*, virulence, environmental selection, natural reservoirs, man-made environments, disease

## Abstract

*Legionella pneumophila* is a ubiquitous bacterium in freshwater environments and in many man-made water systems capable of inducing pneumonia in humans. Despite its ubiquitous character most studies on *L. pneumophila* virulence focused on clinical strains and isolates from man-made environments, so little is known about the nature and extent of virulence variation in strains isolated from natural environments. It has been established that clinical isolates are less diverse than man-made and natural environmental strains, suggesting that only a subset of environmental isolates is specially adapted to infect humans. In this work we intended to determine if unrelated *L. pneumophila* strains, isolated from different environments and with distinct virulence-related genetic backgrounds, displayed differences in virulence, using the Wax Moth *Galleria mellonella* infection model. We found that all tested strains were pathogenic in *G. mellonella*, regardless of their origin. Indeed, a panoply of virulence-related phenotypes was observed sustaining the existence of significant differences on the ability of *L. pneumophila* strains to induce disease. Taken together our results suggest that the occurrence of human infection is not related with the increased capability of some strains to induce disease since we also found a concentration threshold above which *L. pneumophila* strains are equally able to cause disease. In addition, no link could be established between the sequence-type (ST) and *L. pneumophila* pathogenicity. We envision that in man-made water distribution systems environmental filtering selection and biotic competition acts structuring *L. pneumophila* populations by selecting more resilient and adapted strains that can rise to high concentration if no control measures are implemented. Therefore, public health strategies based on the sequence based typing (STB) scheme analysis should take into account that the major disease-associated clones of *L. pneumophila* were not related with higher virulence in *G. mellonella* infection model, and that potential variability of virulence-related phenotypes was found within the same ST.

## Introduction

*Legionella pneumophila* is a facultative intracellular Gram-negative bacterium ubiquitous in both freshwater environments and in many man-made water systems known for its ability to cause disease in humans (Fields, 2008; Newton et al., 2010; Cunha et al., 2016). Eukaryotic phagocytes feed on *L. pneumophila*, so the bacterium's survival and spread relies on the capability to take over the predator cellular mechanism (Fields, 2008; Declerck, 2010; Abdelhady and Garduño, 2013; Richards et al., 2013). Indeed, the machinery underlying *Legionella* virulence is the outcome of a significant selective pressure resulting in a survival mechanism of trained resilience to protozoa phagocytosis (Moliner et al., 2010; Allombert et al., 2013; Richards et al., 2013; Boamah et al., 2017; Oliva et al., 2018).

*L. pneumophila* infection proceeds by a conserved mechanism in unrelated phagocytes, with the formation of a replicative niche, which in turn is a prerequisite for the development of Legionnaires' disease (Isberg et al., 2009; Taylor et al., 2009; Al-Quadan et al., 2012; Hoffmann et al., 2014). In humans, *Legionella* reaches the lungs after inhalation of contaminated aerosol droplets where a parallel procedure allows the bacterium to takeover another phagocyte, lung-based macrophages, leading to infection (Muder et al., 1986; Hubber and Roy, 2010; Newton et al., 2010; Buchrieser, 2011; Finsel and Hilbi, 2015). Recent evidences suggested the rare possibility of person-to-person transmission of *L. pneumophila* under unique environmental conditions (Borges et al., 2016; Correia et al., 2016).

The evolutive dead-end nature of *Legionella* infection in humans implies that the long-term co-evolution with free-living protozoa hosts provides the primary evolutionary pressure for acquisition and maintenance of virulence factors (Moliner et al., 2010; Newton et al., 2010; Coscollá et al., 2011; Luo, 2011; Al-Quadan et al., 2012; Sánchez-Busó et al., 2014). Indeed, protozoa act as a gene pool enabling various microorganisms to evolve by gene acquisition and loss, adapting to an intra-protozoa lifestyle or progress into new forms (Thomas and Greub, 2010; Gomez-Valero et al., 2011; Al-Quadan et al., 2012; Boamah et al., 2017; Oliva et al., 2018). In accordance, recombination, horizontal gene transfer and convergent evolution play an important role (Costa et al., 2010, 2014; Gomez-Valero and Buchrieser, 2013) in assembling a highly plastic *L. pneumophila* genome (Coscollá et al., 2011; Sánchez-Busó et al., 2014).

Regardless of the highly diverse populations of *Legionella* found in natural environments (Veríssimo et al., 1991; Marrão et al., 1993; Costa et al., 2005; Peabody et al., 2017; Zhang et al., 2017; Cassell et al., 2018), *L. pneumophila* serogroup 1 (SG1) is responsible for >84% of the reported cases of disease worldwide (Yu et al., 2002; Harrison et al., 2009; Beauté et al., 2013; Beauté and Network on behalf of the E. L. D. S., 2017). Curiously, the presence of *L. pneumophila* SG1 in the environment (44%) differs significantly from the clinical *L. pneumophila* SG1 strains (84%) (Harrison et al., 2007; Kozak et al., 2009; David et al., 2016). Nevertheless, it remains unclear why specific *L. pneumophila* strains are more connected with disease since it could be related with intrinsic virulence or they could merely be present in great numbers in the environments that support their dispersal to human's (Gomez-Valero et al., 2014; McAdam et al., 2014).

As expected, and despite the ubiquitous character of *Legionella* spp. in fresh water (Fields, 2008; Peabody et al., 2017; Cassell et al., 2018), most studies on the *L. pneumophila* distribution focused on man-made aquatic environments (air conditioning-systems, potable water distribution systems, public fountains, and plumbing fixtures) in an attempt to establish an epidemiological link between putative environmental sources and clinical isolates (Hyland et al., 2008; Nygård et al., 2008; Sanchez et al., 2008; Phin et al., 2014; Hoisington et al., 2015; van Heijnsbergen et al., 2015, 2016). Several studies support that *L. pneumophila* clinical isolates are genetically distinct than man-made and natural environmental isolates (Costa et al., 2005, 2010, 2012, 2014; Harrison et al., 2007, 2009; Coscolla and Gonzalez-Candelas, 2009). These evidences sustain the hypothesis that only a specific subset of environmental isolates are able to cause disease in humans (Harrison et al., 2007; Coscolla and Gonzalez-Candelas, 2009; David et al., 2016). Indeed, virtually nothing is known about the nature and extent of virulence-related variation in *L. pneumophila* strains isolated from natural environments (Costa et al., 2005, 2010, 2014), namely streams, rivers, lakes, boreholes, contrarily to the vast number of virulence-related studies performed with *L. pneumophila* disease-related isolates (Hyland et al., 2008; Nygård et al., 2008; Sanchez et al., 2008; Phin et al., 2014; Hoisington et al., 2015; van Heijnsbergen et al., 2015, 2016).

The use of larvae of the greater Wax Moth *Galleria mellonella* as an infection model has increased in the last years (Ramarao et al., 2012; Cook and McArthur, 2013; Tsai et al., 2016) since mammalian models imply high costs, ethical constrains and special training. *G. mellonella* has been used as an alternative model of infection after establishing a correlation between virulence in insect and mammalian models for a wide range of human pathogens providing useful information on their pathogenicity; namely for *Staphylococcus aureus, Proteus vulgaris, Serratia marcescens, Pseudomonas aeruginosa, Listeria monocytogenes, Streptococcus suis*, or *Enterococcus faecalis* (Junqueira, 2012; Cook and McArthur, 2013; Eisenman, 2015; Champion et al., 2016; Tsai et al., 2016; Velikova et al., 2016; Wojda, 2017). In addition, *G. mellonella* has been reported has as a model for several intracellular bacteria, such as *Burkholderia pseudomallei, Campylobacter jejuni, Francisella tularensis*, and *Coxiella burnetti* (Aperis et al., 2007; Schell et al., 2008; Champion et al., 2010; Wand et al., 2011) which are closely related to *L. pneumophila*. For these reasons, alternative models of infection using insects are increasingly used to characterize the virulence of bacterial strains before passing to animal models.

A major component of the larvae's immunity is the presence of an innate immune system with hemocytes, resembling alveolar macrophages in humans, which *L. pneumophila* is able to infect and replicate making this a suitable model to study pathogenicity (Harding et al., 2012). Furthermore, it was reported that *L. pneumophila* infection in *G. mellonella* is dependent of the strain, infectious dose, growth phase and *dot/icm* presence (Harding et al., 2012, 2013a,b; Aurass et al., 2013; McAdam et al., 2014), making this model suitable to assess putative differences in virulence between *L. pneumophila* strains.

Here we sought to determine if unrelated *L. pneumophila* strains, isolated from different environments and with distinct genetic backgrounds (Chien et al., 2004; Costa et al., 2005, 2010, 2012, 2014) exhibited different levels of virulence using *G. mellonella* infection model. With these data we aimed to elucidate if there is a subset of *L. pneumophila* isolates specially adapted to produce disease and if this feature could be related with their environmental origin. Our results showed that all tested strains caused mortality in *G. mellonella*, in a dose-dependent and strain-specific mode. Nevertheless no link could be established between *L. pneumophila* virulence, environmental origin, and sequence-based typing profile. These data strongly suggest that *L. pneumophila* infection is linked with the ability of some strains to thrive and persist in man-made environments as a result of environmental selection, rather than with their ability to cause disease.

## Materials and methods

### *Legionella pneumophila* strains and *Galleria mellonella* larvae

*L. pneumophila* strains were selected from several environments in order to capture the maximum genetic variability determined in early studies from *dot/icm, sidJ* and T2S related genes (Costa et al., 2010, 2012, 2014). These included 7 isolates from natural environments, 5 isolates from man-made environments, and 4 clinical-related *L. pneumophila* strains (Table 1). From those, 12 belonged to the *L. pneumophila* subsp. *pneumophila*, two to the *L. pneumophila* subsp. *fraseri* and the remaining two strains to the *L. pneumophila* subsp. *pascullei*. *G. mellonella* larvae were obtained from the Institute for Bioengineering and Biosciences, IST, Lisbon, Portugal and stored at room temperature in the dark.

**Table 1:** Characteristics of *L. pneumophila* strains used in this study.

**Strain designation**	**Source**	**Country**	**Subspecies**	**Serogroup**	**Isolation references**
HRD2	Clinical	Portugal (Central)	*L. pneumophila* subsp. *pneumophila*	1	Costa et al., 2010
Lansing3 (ATCC 35251)	Clinical	USA	*L. pneumophila* subsp. *fraseri*	15	Brenner et al., 1988
Los Angeles1 (ATCC 33156T)	Clinical	USA	*L. pneumophila* subsp. *fraseri*	4	McKinney et al., 1979
Philadelphia1 (ATCC 33152T)	Clinical	USA	*L. pneumophila* subsp. *pneumophila*	1	Chien et al., 2004
HUC1	Man-made	Portugal (Central)	*L. pneumophila* subsp. *pneumophila*	1	Costa et al., 2010
IMC23	Man-made	Portugal (Central)	*L. pneumophila* subsp. *pneumophila*	1	Veríssimo et al., 1990
MICU B (ATCC 33735)	Man-made	UK	*L. pneumophila* subsp. *pascullei*	5	Brenner et al., 1988
Por3	Man-made	Portugal (Southern)	*L. pneumophila* subsp. *pneumophila*	1	Costa et al., 2010
U8W (ATCC 33737T)	Man-made	UK	*L. pneumophila* subsp. *pascullei*	5	Brenner et al., 1988
Ice27	Natural	Iceland	*L. pneumophila* subsp. *pneumophila*	1	Costa et al., 2010
Aço12	Natural	Portugal (Azores)	*L. pneumophila* subsp. *pneumophila*	6	Veríssimo et al., 1991
Aço22	Natural	Portugal (Azores)	*L. pneumophila* subsp. *pneumophila*	3	Veríssimo et al., 1991
Aço5	Natural	Portugal (Azores)	*L. pneumophila* subsp. *pneumophila*	6	Veríssimo et al., 1991
Agn2	Natural	Italy (Agano)	*L. pneumophila* subsp. *pneumophila*	1	Costa et al., 2010
Ger10	Natural	Portugal (Northern)	*L. pneumophila* subsp. *pneumophila*	1	Costa et al., 2010
NMex1	Natural	USA (New Mexico)	*L. pneumophila* subsp. *pneumophila*	6	Marrão et al., 1993

### Virulence determination in *Galleria mellonella*

*L. pneumophila* strains were cultured on charcoal-yeast extract (CYE) plates at 37°C for 4 days prior to inoculation into ACES [N-(2-acetamido)-2-aminoethanesulfonic acid]-yeast extract (AYE). For each *L. pneumophila* strain the growth curves were determined in AYE at optical density at 600 nm (OD_600_) to assess the corresponding growth rates in order to confirm that all strains were in post-exponential phase at 21 h, as required for the infection protocol. Additionally, determining *L. pneumophila* CFU/ml values corresponding to an OD6_00_ of 1 allowed to prepare solutions with pre-determined concentrations to be tested (injected) in *G. mellonella* (Table S1). Briefly, for each *L. pneumophila* strain one full loop of bacteria from BCYE plates was suspended in 3 ml of AYE and incubated for 18–19 hours at 37°C with 200 rpm in a shaking incubator. A tube with AYE was used as control to ensure sterility of the media. After incubation, OD_600_ was measured and new inoculums were prepared in 3 ml of AYE to a final OD_600_ of 0.1. Tubes were left in a shaking incubator as described above, this time for 21 h. After this incubation time, OD_600_ was measured again and the inoculum enumerated using the Drop Plate Method to determine the relation between OD_600_ and CFU/ml. According to the predetermined relation between OD_600_ and CFU/ml the injection concentration was normalized for each tested strain.

The infection of *G. mellonella* was performed as previously described (Harding et al., 2012). Briefly, after 21 h of growth, bacteria were diluted in Dulbecco's phosphate-buffered saline (PBS) to OD_600_ corresponding to the required CFU/ml. To be suitable for injection, larvae were at the 5th or 6th instars stage which corresponded approximately to 2–3 cm in length and had no sign of darkening. The injection site (the hindmost left proleg) was disinfected with ethanol and the injection was performed using a micrometer adapted to control the injection volume onto a micro-syringe (Mil-Homens et al., 2010). *G. mellonella* larvae were injected with 10 μl of bacterial suspension prepared and incubated at 37°C in the dark. Ten larvae were injected for each strain of *L. pneumophila* tested. As a control, 10 larvae were injected with D-PBS alone, and 10 untreated insects were included in every experiment. Assays were allowed to proceed for 72 h as pupa formation could occasionally be seen after that (Harding et al., 2012). At least three independent replicates of each experiment were performed. To confirm the injected CFU/ml of *L. pneumophila*, the injected solutions were plated in BCYE plates and enumerated using the Drop Plate Method.

For each *L. pneumophila* strain the injected-larvae were individually examined after 18, 24, 48 and 72 h post-injection (p.i.) for time of death and for several phenotypic characteristics that correlate with different stages of disease (Tsai et al., 2016). Namely, melanization (black, black spots on brown larvae, spots on beige larvae and no melanization), cocoon formation (full cocoon and no cocoon) and movement (move without stimulation, move when stimulated and no movement). The *G. mellonella* Health Index Scoring System (Loh et al., 2013) was used to assesses the larvae health status by assigning scores according to 4 major observations: larvae mobility, cocoon formation, melanization, and survival.

### Statistical analysis

All quantitative data were obtained from at least three independent assays. Standard deviation was used to calculate error bars. The Mantel-Cox test to determine *p*-values for survival and the ordinary one-way ANOVA of Unpaired *t*-test data to determine *p*-values for health index were performed using GraphPad Prism 7.01 software. Differences were considered to be statistically significant if the *p*-value was lower than 0.05.

### *L. pneumophila* allelic profiling

Genotyping was performed according to the 7-gene protocol from the European Working Group for *Legionella* Infections (EWGLI) SBT scheme (http://www.hpa-bioinformatics.org.uk/legionella/legionella_sbt/php/sbt_homepage.php). The seven gene fragments *flaA, pilE, asd, mip, mompS, proA* and *neuA* were amplified and sequenced as previously described (Gaia et al., 2005; Ratzow et al., 2007; Mentasti et al., 2014). Designation of alleles was performed according to the EWGLI SBT database and its combination was represented as an ordered numerical vector. Strain data is summarized in Table 2. The partial sequences of *L. pneumophila flaA, pilE, asd, mip, mompS, proA* and *neuA* genes determined in this study were deposited in GenBank under the accession numbers MG979220 to MG979296.

**Table 2:** Characteristics of *L*. *pneumophila* strains used in this study.

**Isolates used in this study**			***L. pneumophila*** **isolates present in the ESGLI SBT database**								
**Strain designation**	**Source**	**ST^a^**	**N^b^**	**Source (%)**				**Origin**		**Locus variant**	
				**Man made**	**Clinical**	**Natural**	**Unknown**	**Country**	**Continent^c^**	**SLV^d^**	**DLV^e^**
HRD2^#^	Clinical	44	61	9.8	88.5	1.6	–	9	Am, E	4	18
Lansing3 (ATCC 35251)	Clinical	336	2	–	100	–	–	2	Am, As	6	8
Los Angeles1 (ATCC 33156^T^)	Clinical	1,334	6	50	50	–	–	4	Am, As, E	7	9
Philadelphia1 (ATCC 33152^T^)	Clinical	36	61	8.2	91.8	–	–	11	Am, As, E	18	43
IMC23	Man–made	2,370^*^	1	–	100	–	–	1	E	0	0
HUC1^#^	Man–made	2,353^*^	1	100	–	–	–	1	E	3	23
MICU B (ATCC 33735) ^#^	Man–made	1,335	4	100	–	–	–	2	E	0	1
Por3	Man–made	1	1486	43	56	–	0.47	32	Am, As, E,	62	63
U8W (ATCC 33737^T^)	Man–made	1,335	4	100	–	–	–	2	E	0	1
Ice27	Natural	2,334^*^	1	–	–	100	–	1	E	0	0
Aço12	Natural	68	64	56	40	1.6	1.6	13	Am, As, E	19	49
Aço22	Natural	2,333^*^	1	–	–	100	–	1	E	0	4
Aço5	Natural	2,354^*^	1	–	–	100	–	1	E	6	28
Agn2^#^	Natural	2,336^*^	1	–	–	100	–	1	E	1	2
Ger10^#^	Natural	1,362	10	60	30	10	–	5	E	18	93
NMex1^#^	Natural	1,892	2	50	–	50	–	2	Am, As	6	30

a ST, Sequence Type;

b N, number of isolates present in the EWGLI database.

c Am, America; As, Asia; E, Europe;

d SLV, Single Locus Variant;

e DLV, Double Locus Variant;

# Strains significantly different from L. pneumophila Philadelphia 1 type strain on their ability to induce higher G. mellonella mortality;

* *ST reported in this study*.

### Minimum spanning tree based on allelic profiles of *L. pneumophila* STs

The EWGLI SBT database (http://www.ewgli.org/) included data from 11927 isolates (with Sequence Type – ST, attributed), from 63 countries, representing 2467 distinct STs, at the time this analysis was conducted (17.10.2017). The eBURST algorithm (Francisco et al., 2009) implemented in the PHYLOViZ2.0 software (Nascimento et al., 2017) was used to construct a full minimum spanning tree (MST) displaying the relationships between the allelic profile of the studied strains and other STs described worldwide since groups are all believed to be descended from the same founding genotype (the primary founder) (Correia et al., 2016). STs that couldn't be assigned to any group were called singletons. The statistical confidences for the founders were assessed using 1000 bootstrap re-samplings. Clustering of ST profiles from the studied strains was determined by calculating the Hamming distance using the Unweighted Pair Group Method with Arithmetic Averages (UPGMA), implemented in the PHYLOViZ2.0 software (Nascimento et al., 2017).

### STs clustering and phylogenetic analysis

The similarity between the ST profiles was determined by calculating the Pearson's coefficient with correction: d = (1 - r) x 100. Allelic profile were clustered with the Unweighted Pair Group Method with Arithmetic Averages (UPGMA), employing the DendroUPGMA computer program (http://genomes.urv.cat/UPGMA/) (Garcia-Vallvé and Palau, 1999). Phylogenetic analyses were performed using MEGA5 package (Tamura et al., 2011). Alignment against the corresponding genes found in the EWGLI SBT database for the *L. pneumophila* strains included in this analysis (Table 1), was performed using the multiple alignment CLUSTAL software (Higgins, 1994), included on MEGA5 package. Maximum likelihood phylogenetic tree was obtained for the concatenated alignment with PhyML 3.0 (Guindon and Gascuel, 2003), using the most appropriate model of nucleotide substitution and likelihood scores assessed by TOPALi V2.5 (Milne et al., 2004) and by jModeltest (Posada, 2008). The best model was determined by using the Akaike Information Criterion (AIC) (Akaike, 1974; Posada and Buckley, 2004). Supports for the nodes were evaluated by bootstrapping with 1000 pseudoreplicates.

## Results

### Mortality of *G. mellonella* infected with diverse isolates of *L. pneumophila* is dose dependent

Determining *L. pneumophila* CFU/ml corresponding to an OD_600_ of 1 for each of the tested strains was mandatory to prepare suspensions for injection with a pre-determined concentration in order to standardize the experiment. Differences between the CFU/ml values that corresponded to an OD_600_ of 1 were observed between strains. The most notorious difference was observed for strain Por3 with a CFU/ml value of 2.6 x 10^8^, one order of magnitude lower than the one recorded of all other strains (Table S1). These results were taken into account in order to guarantee that the concentration of injected bacteria was the same for all *L. pneumophila* strains.

As previously mentioned, one of the goals of this study was to determine if there were differences between *L. pneumophila* strains regarding their capability to induce larvae mortality. To answer this question, three different injection concentrations (10^5^, 10^6^ and 10^7^ CFU/larvae) were tested in *G. mellonella* in three *L. pneumophila* strains. This allowed us to select the injection concentration that showed greater differences on larvae survival to be used in the following experiments.

From the tested conditions, no mortality was observed for the 10^5^ CFU/larvae injection concentration within the experimental time course, similar to what was observed for the D-PBS injected larvae, serving as control (Figure S1a).

Differences on larvae survival between strains were observed after injection with 10^6^ CFU/larvae. In the first 24 h p.i., 100% of larvae survival was registered for all strains. The differences between strains were more pronounced with time. Namely, for IMC23 and Philadelphia1 a 20% larvae mortality occurred at 48 h p.i., while for strain Aço5 this value was 10%. In addition, Philadelphia1—infected *G. mellonella* had a larvae mortality of 60% after 72 h p.i. while for strains IMC23 and Aço5 the mortality values were of 80%. Nevertheless, we did not observe 100% of mortality on larvae injected with this concentration. Similarly, no mortality was observed for the D-PBS injected larvae serving as control (Figure S1b).

At the highest concentration no major differences on larvae survival were observed between strains since all induced a larvae mortality of 70, 90, and 100% at 24, 48 and 72 h p.i., respectively. Nevertheless, the survival curve differed between strains at 18 h p.i., where strain Philadelphia 1 induced a larvae mortality of 20% while strain Aço5 and IMC23 induced 40% of larvae mortality. No mortality was observed for the D-PBS injected larvae serving as control (Figure S1c).

Ultimately, differences in larvae survival were observed between the three injection concentrations since *G. mellonella* was able to survive low infectious dose but succumbed to higher doses. These observations confirmed that *L. pneumophila* virulence in *G. mellonella* was dose dependent (Harding et al., 2012). As infection with 10^7^ CFU/larvae caused very rapid death, any differences in virulence of the tested strains would be disguised. Hence, in the following experiments, we injected larvae with an infectious dose of 10^6^ CFU in order to identify differences on virulence between strains.

### *L. pneumophila* virulence in *G. mellonella* was strain and time dependent

Prior work evidenced that three usually studied SG1 *L. pneumophila* strains caused death of at least, 70% of *G. mellonella* larvae accompanied by severe organ damage translated in larvae melanization. These results were dependent of the strain, infectious dose, growth phase and presence of T4SS (Harding et al., 2012). We intended to corroborate those evidences and broader the overall picture by using a wider group of unrelated *L. pneumophila* strains, isolated from different environments and with distinct genetic backgrounds (Chien et al., 2004; Costa et al., 2005, 2010, 2012, 2014) (Table 1). Briefly, to determine the intrinsic capability of *L. pneumophila* strains to induce mortality in *G. mellonella*, 10 larvae were injected with 10^6^ CFU/larvae of each tested strain and their survival was monitored for 72h (Table S2). A control group was also tested by injection with 10 μl of D-PBS.

**Figure 1:**
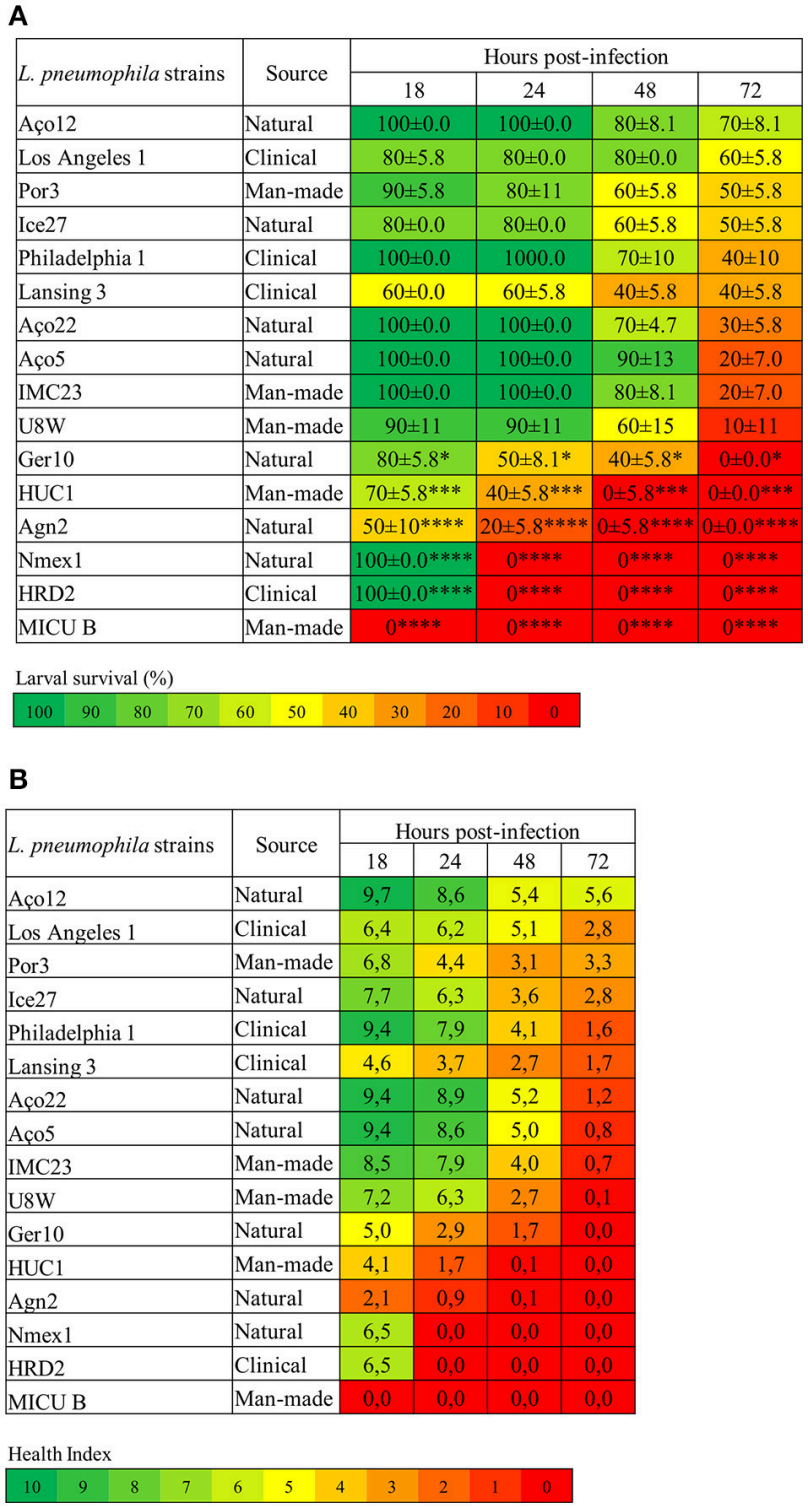
*L. pneumophila* virulence in *G. mellonella* is strain-specific. **(A)** Larvae survival (%) **(B)** Health Index. *G. mellonella* larvae were infected with 10^6^ CFU of each of the tested strains or D-PBS, and the survival rate and health status was monitored for 18, 24, 48, and 72 h. Injection with D-PBS was used as control and no mortality was observed (data not shown). *L. pneumophila*-induced mortality in *G. mellonella* was dependent on the tested strain with significant differences (*P* < 0.0001) between strains. In addition for the larvae survival, each strain was also statistically compared with *L. pneumophila* Philadelphia 1 and differences were considered significant if *P < 0.05, highly significant if ***P < 0.001 and extremely significant if ****P < 0.0001. Results are the mean of at least three independent experiments; ± standard deviation.

Foremost, *L. pneumophila* strains demonstrated significantly (*P* < 0.0001) differences on the capability to induce *G. mellonella* mortality (Figure 1A). Indeed, some strains induced 100% larvae mortality while others only induced some mortality, although at distinct rates (Figure 1A). Namely, strain MICU B induced 100% larval mortality after 18 h p.i., while NMex1 and HRD2 showed the same results after 24 h p.i. Agn2 and HUC1 strains also induced 100% larval mortality but in this case after 48 h p.i. Lastly, Ger10 hit 100% mortality after 72 h p.i. In contrast, some larvae survival was seen for the remaining strains despite relevant differences on the infection kinetics (% induced larvae mortality); namely for strains U8W (90%), IMC23 and Aço5 (80%), Aço22 (70%), Philadelphia 1 and Lansing3 (60%), Ice27 (50%) and Los Angeles1 (40%) (Figure 1A). Nevertheless they all had a common characteristic, all tested strains induced death at a given point.

These results allow us to conclude that larvae mortality kinetics and infection profile were strain specific. Moreover, a panoply of virulence-related phenotypes were observed as some of the tested strains were defective in their ability to cause disease; while others were highly pathogenic. Since a correlation has been established between the virulence variation in *G. mellonella* model and the virulence variation observed in mammalian cell culture and animal models for several human pathogens (Ramarao et al., 2012; Cook and McArthur, 2013; Champion et al., 2016; Tsai et al., 2016; Velikova et al., 2016; Barnoy et al., 2017) altogether our data suggest the existence of potential differences on the ability of *L. pneumophila* strains to induce disease in humans.

### All *L. pneumophila* strains were pathogenic in *G. mellonella*

In order to detect more subtle differences between *L. pneumophila* strains ability to cause disease, the *G. mellonella* Health Index Scoring System was used (Loh et al., 2013; Tsai et al., 2016). Briefly, each larvae was simultaneously scored for the survival rate (Figure 1A) and health status (Figure 1B), through the observation of several features; namely activity, cocoon formation and melanization, that correlates with different stages of disease. As previously described, higher activity and cocoon formation correspond to a healthier larvae. Melanization refers to the synthesis and deposition of melanin that occurs as a result of an immune response against infection, as melanin aids to encapsulate and kill pathogens. This process usually starts with distinctive black spots that increase in size followed by the complete melanization (black larvae) that correlates with death of the larvae soon after (Loh et al., 2013). A score was assigned to each observation and an overall health index score was calculated for each larvae, ranging from 10 (healthy larvae) to 0 (dead larvae) (Tsai et al., 2016) (Figure 1B and Table S2).

Decreasing health index scores relates well with increasing virulence of *L. pneumophila* strains (Figure 1), and it was clear that all strains induced morphologic alterations in the infected larvae at a certain point. Indeed, larvae injected with the most virulent strains showed a rapid decline in health that matched the survival rate. Remarkably, even in strains that induced partial larvae mortality, the survivors displayed reduced health index values (Figure 1B). This reinforced that all tested strains were pathogenic in *G. mellonella*.

Nevertheless, despite strains Aço12 and Por3 induced respectively 30% and 50% larval mortality at the end of the experiment, the survivor's showed signs of recovery between 48 and 72 h p.i. based on the health index (Table S2). Observing the data from morphological characteristics regarding these strains, it was noticed that until 48 h p.i. larvae presented common alterations, such as the increase of darkening, no cocoon formation and slow movement. Nevertheless, between 48 and 72 h p.i., larvae showed signs of recovery. Indeed, the number of dark larvae stabilized or even decreased with a concomitant increase in the number of normal color larvae. The same happened for cocoon formation and movement. These observations suggested that some larvae could partially overcome infection with *L. pneumophila* strains Aço12 and Por3 (Figure 1B). Since *L. pneumophila* strains used in this work had distinct virulence-related genetic repertoire (Costa et al., 2010, 2012, 2014), one possible explanation for the above-mentioned results could be related with these strains genetic context translated in a limited ability to replicate or persist in the larvae.

### The origin of *L. pneumophila* strains does not correlate with virulence in *G. mellonella*

As previously mentioned, studies supported the premise that only a specific subset of environmental *L. pneumophila* strains were actually capable of producing infection in humans (Coscolla and Gonzalez-Candelas, 2009; David et al., 2016). In line, our above-mentioned results corroborate that *L. pneumophila* strains demonstrated statistically significant (*P* < 0.0001) differences on their ability to induce *G. mellonella* mortality (Figure 1). To further investigate this hypothesis statistical analysis was performed to determine if the pathogenicity of *L. pneumophila* in *G. mellonella* was related with the origin of the strains since nothing is known about the nature and extent of virulence variation in isolates from natural environments. This is the first report in which natural environmental *L. pneumophila* strains were tested for their ability to cause infection.

Regarding strains isolated from natural environments, significant differences (*P* < 0.0001) were observed on their ability to kill larvae. For instance, strain NMex1 induced 100% of larvae mortality 24 h p.i. while strain Aço12 only induced 30% of larvae mortality and in a longer period of time (72 h p.i.) (Figure 1A). Similarly, significant differences (*P* < 0.0002) were observed on the ability of man-made environmental strains to kill larvae. Namely, strains MICU B and HUC1 were capable of inducing 100% of larvae mortality, while Por3 only induced 50% of larvae mortality within the experiment time frame (Figure 1B). Regarding the clinical-related strains, a *P* < 0.0001 supported significant differences among strains, as previously observed for other clinical strains (Harding et al., 2012). Namely, HRD2 induced 100% of larvae mortality at 24 h p.i., while Los Angeles 1 was only capable of causing 40% of larvae mortality at 72 h p.i. (Figure 1A). Decreasing health index scores relates well with increasing virulence of *L. pneumophila* strains (Figure 1), and it was clear that all strains induced morphologic alterations in the infected larvae at a given point.

Our results show that *L. pneumophila* pathogenicity is not related with the origin of the strains. In fact, we were able to identify strains capable of inducing 100% of mortality in *G. mellonella* isolated from all environmental types. Likewise, strains with a reduced ability to cause death were also isolated from all environmental types (Figure 1).

The genus *Legionella* comprises over 60 species, being *L. pneumophila* the leading cause of Legionnaire's Disease (Khodr et al., 2016). Based on DNA-DNA hybridization tests *L. pneumophila* was classified into three subspecies, *L. pneumophila* subsp. *pneumophila, L. pneumophila* subsp. *fraseri* and *L. pneumophila* subsp. *pascullei* (Brenner et al., 1988). *L. pneumophila* subsp. *pneumophila* has been associated with almost 85–90% of the disease cases worldwide (David et al., 2016). Nevertheless, it has not been possible to establish a correlation between this incidence and their environmental persistence or virulence (Doleans et al., 2004; Harrison et al., 2007; Kozak et al., 2009; David et al., 2016). In order to determine if the aforementioned predominance of *L. pneumophila* subsp. *pneumophila* in disease cases could be related with higher virulence, the pathogenicity of the three *L. pneumophila* subsp. was analyzed. All subsp. caused time-dependent death of at least 40% of the *G. mellonella* larvae; in fact *L. pneumophila* subsp. *pascullei* strain MICU B caused significantly (*P* < 0.005) higher mortality than *L. pneumophila* subsp. *pneumophila* strains, 18 h p.i. Considering the link previously determined between the virulence of several human pathogens in *G. mellonella* and their ability to cause disease (Cook and McArthur, 2013; Tsai et al., 2016), these results suggest that some *L. pneumophila* strains are more frequently related with human disease, not because they are more virulent, but most probably because of their wider environmental distribution, higher concentration or ability to persistence in the man-made environments.

### Comparison between the ability of *L. pneumophila* type strain philadelphia 1 and the other tested strains to infect *G. mellonella*

Philadelphia 1 is the type strain of *L. pneumophila* subsp. *pneumophila* (SG1) and is derived from the original clinical isolate collected during the first recognized outbreak of Legionnaires' disease (Fraser et al., 1977; McDade et al., 1977). This strain has been used in numerous laboratories worldwide and was the basis of the research conducted on this important human pathogen (Chien et al., 2004; Gomez-Valero and Buchrieser, 2013; Cunha et al., 2016). Since the capability of Philadelphia 1 to infect *G. mellonella* has already been tested by others (Harding et al., 2012), we decided to use it as a reference strain for comparison purposes. This strategy allowed us to ascertain if there were, or not, significant differences on the ability to kill larvae between strains recovered from other environments, namely natural and man-made environments, in opposition to clinical-related strains.

Indeed, significant differences were observed on the ability of *L. pneumophila* strains to induce larvae mortality when compared to Philadelphia 1 (Figure 1). Surprisingly, the aforementioned strains included natural environmental isolates, namely Agn2 and NMex1 (*P* < 0.0001) and Ger10 (*P* < 0.05), and as expected clinical-related and man-made environmental isolates, namely HRD2 and MICU B (*P* < 0.0001) and HUC1 (*P* < 0.001). All other strains exhibited a behavior that was not significantly different from the reference strain (Figure 1).

Since Philadelphia 1 has been responsible for several outbreaks of Legionnaire's Disease (Muder and Yu, 2002; Beauté et al., 2013; David et al., 2016; Beauté and Network on behalf of the E. L. D. S., 2017), the identification of strains with the same capability to induce larvae mortality may suggests that they could have identical potential to cause disease in humans. Moreover, those strains were isolated from both natural and man-made environments, contradicting the hypothesis that only a specific subset of environmental isolates is actually capable of producing infection in humans (Coscolla and Gonzalez-Candelas, 2009; Harrison et al., 2009; David et al., 2016). Our data reinforces that the occurrence of human infection may not be related with the increased capability of some strains to induce disease. In fact, the decisive factor is probably linked with *Legionella* ability to persist and multiply to high numbers in man-made environments that generate contaminated aerosols that might be dispersed over a wide area.

### Relation between the studied strains and the worldwide *L. pneumophila* genetic diversity inferred from SBT

An internationally recognized seven-gene molecular typing scheme—Sequence-Based Typing (SBT), has been developed for genotyping *L. pneumophila* isolates (Gaia et al., 2005; Ratzow et al., 2007) and a curated database has been established by The European Working Group for *Legionella* Infections (EWGLI). The SBT analysis was performed to ascertain whether there was a correlation between *L. pneumophila* Sequence Types (STs), source and virulence. Data from reference and type strains was retrieved from the EWGLI SBT database and compared with the STs obtained for the strains used in this study. The results showed that the 16 analyzed strains matched 15 STs. In total, 10 strains clustered in STs previously reported in the EWGLI SBT database, namely Por3 (ST1), Philadelphia1 (ST36), HRD2 (ST44), Aço12 (ST68), Lansing3 (ST336), Los Angeles 1 (ST1334), MICU B and U8W (ST1335), Ger10 (ST1362), and NMex1 (ST1892). The remaining six isolates (37.5%) displayed newly reported STs, namely Aço22 (ST2333), Ice27 (ST2334), Agn2 (ST2336), HUC1 (ST2353), Aço5 (ST2354), and IMC23 (ST2370) (Table 2). Only *L. pneumophila* subsp. *pascullei* strains U8W and MICU B exhibited the same SBT profile.

**Figure 2:**
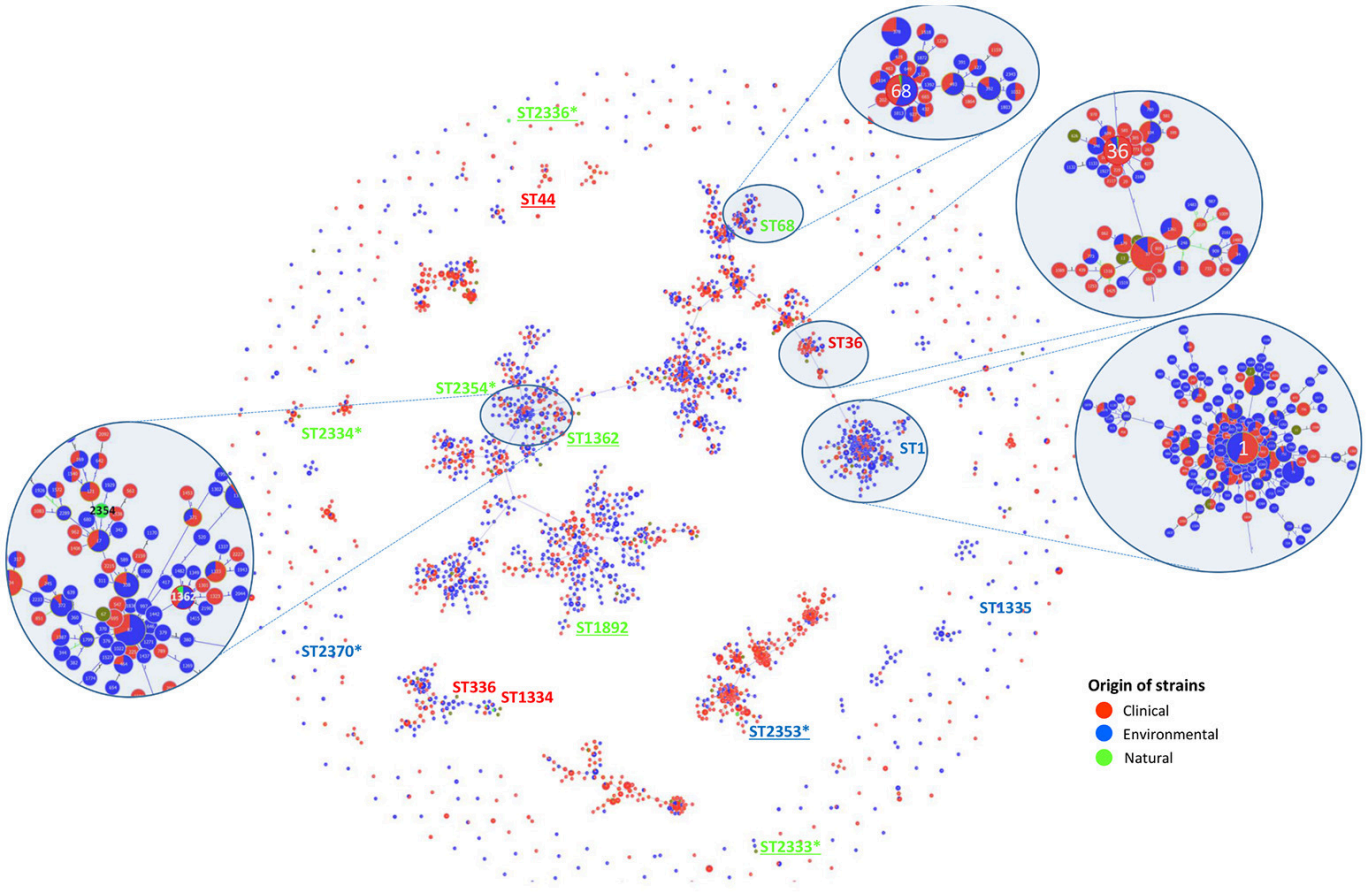
SBT-based representation of the worldwide genetic diversity among *L. pneumophila* isolates. Relationships between 2,342 allelic profiles of 11,737 isolates (from the EWGLI SBT database) are shown in a goeBURST full minimum spanning tree (MST). The MST connects the ST profiles in a way that the summed distance of all links of the tree is the minimum. STs found in this work are identified. Pies represent the different STs with the size being proportional to haplotype frequencies. The source of the isolates is color coded: clinical-related (red), man-made environment (blue), natural environment (green), and unknown (brown). Underlined strains were significantly different from *L. pneumophila* Philadelphia 1 type strain on their ability to induce higher *G. mellonella* mortality; *New ST described in this study.

To establish genotypic relationships and possible correlations with the source of the strains, a cluster analysis at single and double locus variant level was performed by goeBURST algorithm (Figure 2). All strains clustered in a ST group, with the exception of Ice27 (ST2334) and IMC23 (ST2370), which were singletons. Among the 15 STs present in this study, only five had more than 10 isolates in the EWGLI database, namely ST1, ST68, ST44, ST36, and ST1362 (Table 2).

The man-made environmental isolate Por3 matched ST1 (also known as the “Paris-like strain”), detected among 1.468 isolates with a worldwide distribution (32 countries from 5 continents) (Table 2). Recent reports revealed that ST1 belongs to a subset of five STs (1, 23, 37, 47, and 62) that are responsible for nearly half of all epidemiologically unrelated cases of Legionnaire's disease (David et al., 2016). Interestingly, from those, only ST1 has been equally isolated from environmental and clinical sources, while the remaining four STs were mostly related with disease (David et al., 2016).

Strain Aço12 was assigned to the widely dispersed ST68 (13 countries from 3 continents) along with 64 strains in the database. Interestingly, no linkage could be established between this ST and the strain source since the isolates have been equally recovered from clinical and environmental sources (Figure 2 and Table 2).

On the other hand, a predominance of clinical isolates (92%) was clear for the globally disperse ST36, comprised of *L. pneumophila* type strain Philadelphia 1 and 60 other isolates (11 countries and 3 continents). The same trend was observed for ST44, since more than 88% of the strains were clinical, regardless its broad distribution (9 countries from 2 continents). The clinical-related strain HRD2 was assigned to this ST along with 60 other strains. Additionally, the natural environmental strain Ger10 belonged to ST1362, comprising predominantly environmental isolates (70%), recovered from 5 European countries. This is a restrict group with only 10 strains (Figure 2 and Table 2).

Four strains from *L. pneumophila* subsp. *fraseri* and *pascullei* were also included in this analysis, corresponding to three distinct ST profiles. *L. pneumophila* subsp. *fraseri* type strain Los Angeles 1 was included in ST1334, along with 6 other isolates from both environmental and clinical sources (50% each), from 4 different countries in 3 continents. On the other hand, just one other strain matched ST336 along with strain Lansing3, both from clinical samples collected from two continents. The two strains from subsp. *pascullei* had the same ST1335 profile along with two other environmental isolates from Europe (Figure 2 and Table 2).

These results were in accordance with our initial goal to use unrelated strains. Moreover, the newly reported STs were identified from environmental strains confirming the frequently reported bias on *L. pneumophila* diversity due to the over representation of clinical-related strains (Hyland et al., 2008; Nygård et al., 2008; Sanchez et al., 2008; Phin et al., 2014; Hoisington et al., 2015; van Heijnsbergen et al., 2015, 2016). In addition, several natural environmental strains clustered in clonal complexes along with clinical strains known to cause disease in humans. Since the described natural environmental strains were equally pathogenic in *G. mellonella* model, one could envision a similar trend in humans. Nevertheless, further studies will be necessary to prove this fate in humans.

### Relation between *L. pneumophila* ST, strain origin and ability to induce mortality in *G. mellonella*

**Figure 3:**
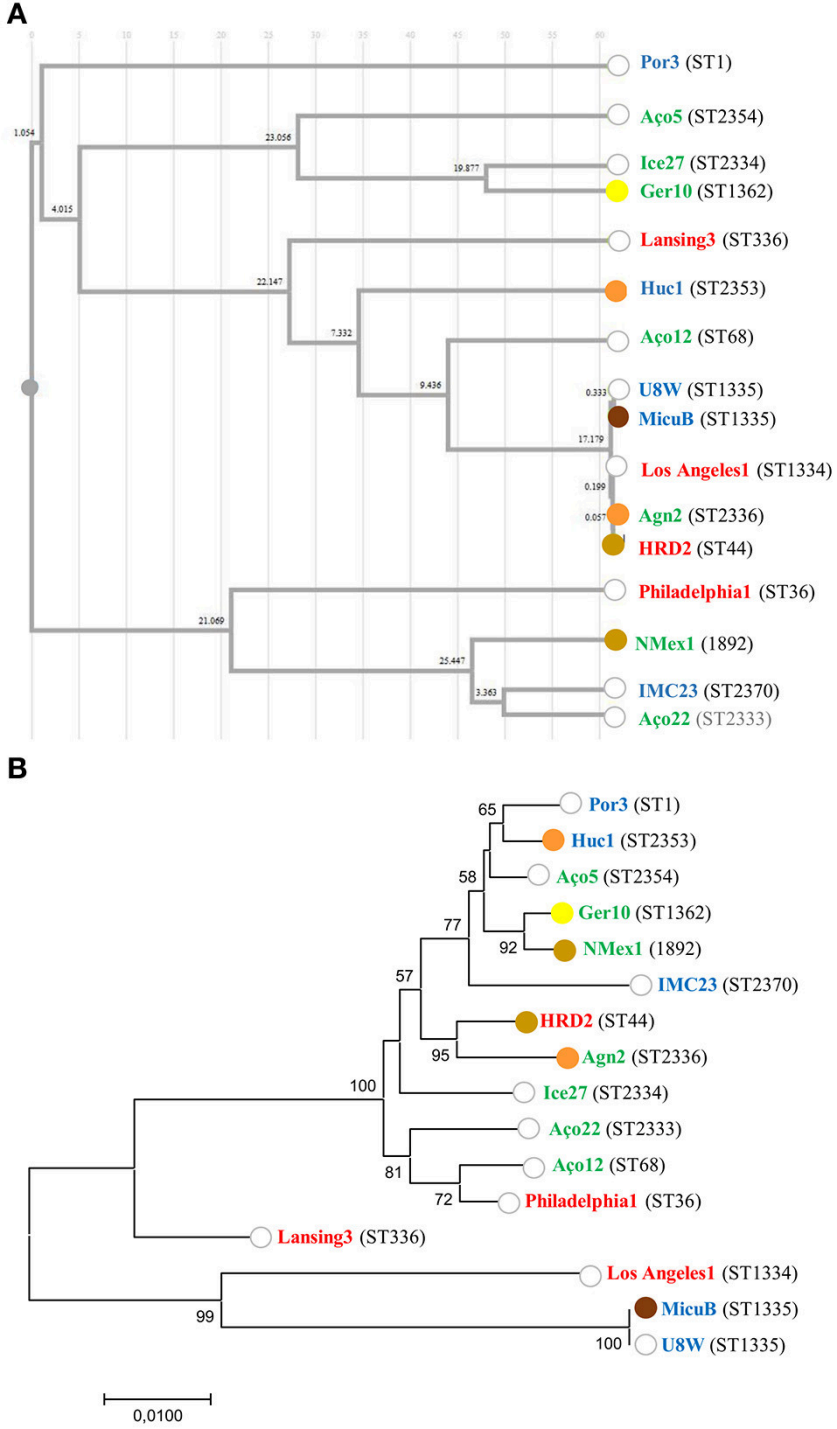
Relation between *L. pneumophila* ST, strain origin and ability to induce mortality in *G. mellonella*. **(A)** Cluster analysis dendrogram inferred from the SBT profiles based on the UPGMA algorithm with Pearson correlation coefficient implemented in DendroUPGMA; **(B)** Maximum likelihood tree from the concatenated alignment of the nucleotide sequences from the SBT analysis. Bootstrap support values (1,000 replicates) for nodes higher than 50% are indicated. The ability to induce 100% mortality in *G. mellonella* over time is color coded: 18 h (brown); 24 h (light brown); 48 h (orange); 72 h (yellow); do not reach 100% mortality (white). The source of the isolates is color coded: clinical-related (red), man-made environment (blue), natural environment (green).

In order to capture the relationship between strains inferred from the SBT analysis, an Unweighted Pair Group Method with Arithmetic Averages (UPGMA) was used to build a dendrogram (Figure 3A). The method calculates all the Pearson correlation coefficients between pairs of sets of variables, transforms these coefficients into distances and makes a clustering using the UPGMA algorithm (Garcia-Vallvé and Palau, 1999). These results allow us to conclude that the ST profiles did not reconstruct *L. pneumophila* species phylogeny, since *L. pneumophila* strains from the same sub-species were in distinct clusters with strains from other subsp., namely Lansing 3 and Los Angeles 1 (*L. pneumophila* subsp. *fraseri*). This was also supported by the phylogeny inferred from the concatenated alignment of the SBT genes since no relation was observed between the distribution of the strains and virulence (Figure 3B). Additionally, no relation could be established between ST's and the origin of strains since isolates from distinct environments were grouped, while strains from the same environmental type were distributed by distinct clusters (Figure 3A).

Previous reports assumed that only a specific subset of *L. pneumophila* environmental isolates were actually capable of producing infection in humans (Harrison et al., 2007; Coscolla and Gonzalez-Candelas, 2009; David et al., 2016). Our abovementioned results obtained in *G. mellonella* model did not support this hypothesis since all *L. pneumophila* strains tested were pathogenic (Ramarao et al., 2012; Cook and McArthur, 2013; Champion et al., 2016; Tsai et al., 2016; Velikova et al., 2016; Barnoy et al., 2017). Nevertheless, our findings raised another hypothesis: could a sub-set of *L. pneumophila* strains belonging to globally distributed ST's and more often related with clinical cases be more virulent? In order to confirm this hypothesis, we compared the ability of unrelated strains to infect *G. mellonella* based on their ST (Figure 3A). From our results we were not able to establish a linkage between STs and *L. pneumophila* pathogenicity. In fact, the most virulent strains in *G. mellonella* model (Figure 1) were included in new STs or in STs with very few isolates, with the exception of strain HRD2 (Figure 2 and Table 2). In other words, the STs most commonly associated with clinical cases and widely distributed, namely ST1, ST36, ST336, were represented in this study by strains with an average ability to infect *G. mellonella* (Figure 3A). Interestingly, the less pathogenic strain assessed in this study, the natural environmental strain Aço12, was clustered in ST68, along with another 64 isolates from man-made environments and clinical cases. Finally, we could not correlate the SBT types, or SBT-based clusters, with the degree of virulence since strains capable of inducing similar results regarding larvae mortality were in separate clusters. Likewise, strains with distinct virulence patterns were clustered (Figure 3A).

The SBT scheme has been used for several years for epidemiological typing of clinical and environmental isolates but we couldn't find any link between pathogenicity and STs. In order to determine if strains belonging to the same ST had the same ability to induce disease we analyzed ST355. This ST comprised two strains of *L. pneumophila* subsp. *pascullei* that exhibited significant differences on their pathogenicity since MICU B induced 100% larvae mortality 18 h p.i. while U8W did not reach 100% of larvae mortality during the time frame of the experiment (Figure 1). This indicate that some STs are heterogeneous since they comprise strains with distinct ability to cause disease. In sum, public health strategies based on the SBT scheme analysis should take into account that the major disease-associated clones of *L. pneumophila* are not associated with higher virulence and that potential variability of virulence-related phenotypes may be found within the same ST.

## Discussion

Given the evolutive dead-end nature of *L. pneumophila* infection in humans the study of the pathogenicity of *L. pneumophila* from natural environments isolates, as well as from man-made environments and clinical samples, is important to determine which factors contribute to the occurrence of disease. Using seven isolates from natural environments, five from man-made environments and four clinical-related *L. pneumophila* strains, we found that all strains prove to be pathogenic in *G. mellonella* infection model. In accordance with previous studies (Harding et al., 2012), larvae mortality kinetics and infection profile were strain specific. Indeed, a wide variety of virulence-related phenotypes were observed. While some of the tested strains were defective in their ability to cause disease others were highly pathogenic. Since the *L. pneumophila* isolates used in this study had distinct virulence-related genetic repertoires (Costa et al., 2010, 2012, 2014) a possible explanation for the above mentioned results could be related to differences in the strains ability to replicate or persist in the infected larvae. Altogether our data could substantiate the existence of significant differences on the ability of *L. pneumophila* strains to induce disease since a correlation has been established between the virulence variation of several human pathogens in *G. mellonella* model, mammalian cell culture and animal models (Ramarao et al., 2012; Cook and McArthur, 2013; Champion et al., 2016; Tsai et al., 2016; Velikova et al., 2016). The whole genome sequencing of these *L. pneumophila* natural environmental strains will allow performing genome-wide association studies (GWAs) that will clarify if these variables are statistically dependent. In fact, several GWAs performed in other human pathogens in which differences in genotype frequencies were compared between cases and controls identified novel features related with virulence (Chen and Shapiro, 2015; Omae et al., 2017; Yang et al., 2018).

When *L. pneumophila* pathogenicity was compared with the origin of the strains no correlation could be established. Indeed, we identified strains capable of inducing 100% of mortality in *G. mellonella* isolated from all environmental types. Likewise, strains with a reduced ability to cause death were also isolated from different environments. These findings contradict the idea that isolates of *L. pneumophila* recovered from clinical cases and man-made environments represent a limited, non-random subset of all genotypes existing in nature with increased capability to induce infection in humans (Harrison et al., 2007; Coscolla and Gonzalez-Candelas, 2009; Costa et al., 2010; David et al., 2016). In addition, we determined that there is a concentration threshold above which *L. pneumophila* strains are equally able to cause disease in *G. mellonella*. Overall, our data strongly suggests that the ability of some *L. pneumophila* strains to cause disease is more likely related with their capability to persist and thrive in man-made environmental niches, which are linked with human infection, and not related with their virulence. This idea is in line with previous reports proposing that natural environments are possible sources or reservoirs of *Legionella* sp. impacting the spatial distribution of sporadic cases of disease (Ortiz-Roque and Hazen, 1987; Rocha et al., 1995; Costa et al., 2005; Peabody et al., 2017; Cassell et al., 2018).

It is well-documented that the intracellular growth within protozoa is the prevailing mechanism of legionella proliferation (Rowbotham, 1986; Fields, 1996; Boamah et al., 2017; Oliva et al., 2018). Moreover, recent reports determined that the distribution of protozoa between different environmental reservoirs is relatively constant (Boamah et al., 2017). Nevertheless, not all protozoa species are hosts of *L. pneumophila*. Thus, the relative abundance of *L. pneumophila* in different environmental niches may be related with variations in the heterogeneous populations of protozoan hosts and non-hosts (Declerck, 2010; Amaro et al., 2015). Indeed, several factors affect the outcome of legionella-protozoa interaction, namely the genetic composition of the bacterium since dramatic differences have been reported between strains and species (host tropism), the relative abundance of each organism and the external environment (García et al., 2007; Declerck, 2010; Buse and Ashbolt, 2011; Boamah et al., 2017). Several studies determined that the structure and dynamics of microbial communities may be influenced by abiotic environmental filtering and biotic competition (Winter et al., 2013; Heino et al., 2014; Zhou et al., 2017). We propose that this vision should be applied to the *L. pneumophila* pathway from natural reservoirs to human infection. Is well-known the distinct distribution pattern of legionellae between reservoirs. So, the ability of legionellae to reside, survive and multiply in biofilm communities that are known to provide protection and nutrients (Taylor et al., 2009; Declerck, 2010; Abdel-Nour et al., 2013; Vatansever and Türetgen, 2015) could represent crucial features for selection by environmental filtering and competition explaining the predominance of some disease-related strains. Indeed, despite the presence of diverse *Legionella* sp. in natural aquatic reservoirs (Veríssimo et al., 1991; Marrão et al., 1993; Costa et al., 2005; Peabody et al., 2017; Zhang et al., 2017; Cassell et al., 2018), *L. pneumophila*, namely SG1, is responsible for the vast majority of the reported cases of disease worldwide (Yu et al., 2002; Harrison et al., 2009; Beauté et al., 2013; Beauté and Network on behalf of the E. L. D. S., 2017). However, the environmental distribution of *L. pneumophila* SG1 (44%) differs significantly from the distribution of clinical *L. pneumophila* SG1 strains (Harrison et al., 2007; Kozak et al., 2009; David et al., 2016). In our opinion, environmental filtering and biotic competition represent the most probable evolutive constrains structuring the diversity of *L. pneumophila* found in artificial environments aiding to explain our results.

Transmission of Legionnaires' disease usually occurs by inhalation of aerosols or aspiration of water containing *Legionella* spp. from point-sources that dispersed contaminated aerosols over a wide area (Mercante and Winchell, 2015). In these chain of events the concentration of Legionella in water systems is a key factor in terms of greatest threat to public health and one should not rely solely on the presence, virulence of the strain and population health status. Indeed our results demonstrate the existence of a concentration threshold above which *L. pneumophila* strains are equally able to cause disease. This implies that the presence of *L. pneumophila per si* is not synonymous of a threat to public health but reinforces the need to carefully manage the concentration of this bacterium in water distribution systems to avoid reaching a critical threshold. In this way, it is crucial for water distribution systems management and public health to further co-relate *Legionella* concentration with the probability to cause disease in countries where appropriate surveillance is in place.

## Author contributions

LM, AV, and JC designed the study. PS and IS were in charge of laboratory procedures. PS did the statistical analysis. AV and JC wrote the manuscript. All authors read and approved the final manuscript.

### Conflict of interest statement

The authors declare that the research was conducted in the absence of any commercial or financial relationships that could be construed as a potential conflict of interest.
