# Supplementary figures and images for: Differences in Virulence Between Legionella pneumophila Isolates From Human and Non-human Sources Determined in Galleria mellonella Infection Model
Source: Front Cell Infect Microbiol. 2018 Apr 4;8:97. doi: 10.3389/fcimb.2018.00097 (PMC5893783; doi:10.3389/fcimb.2018.00097)

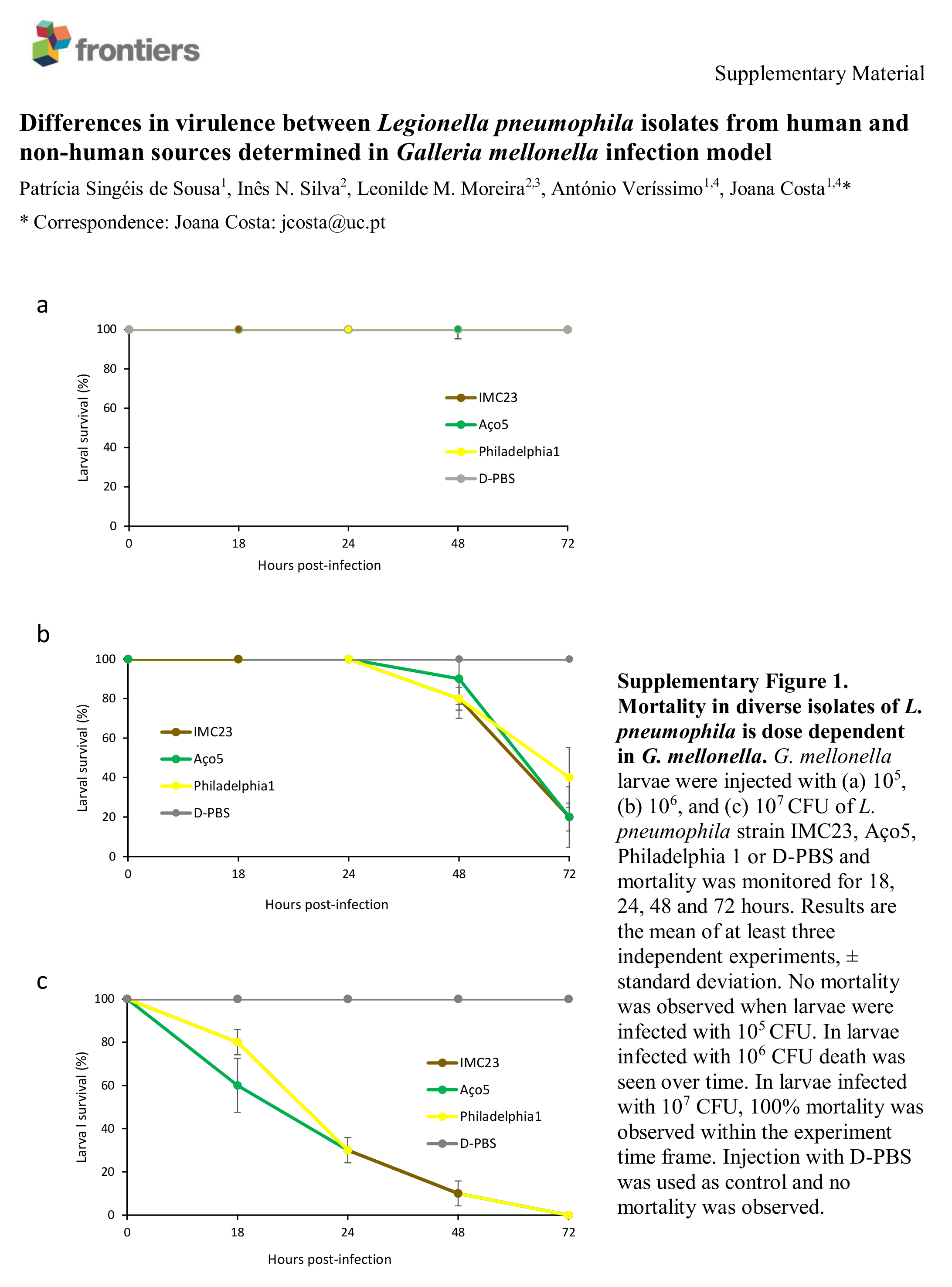

Supplement: Supplementary file 1 [file Image1.TIF]
