# Supplementary material for: Differences in Virulence Between Legionella pneumophila Isolates From Human and Non-human Sources Determined in Galleria mellonella Infection Model
Source: Front Cell Infect Microbiol. 2018 Apr 4;8:97. doi: 10.3389/fcimb.2018.00097 (PMC5893783; doi:10.3389/fcimb.2018.00097)
Supplement: Supplementary file 2 [file Table1.PDF]

Differences in virulence between *Legionella pneumophila* isolates from human and non-human sources determined in *Galleria mellonella* infection model

Patrícia Singéis de Sousa[1], Inês N. Silva[2], Leonilde M. Moreira[2,3], António Veríssimo[1,4], Joana Costa[1,4]*

* Correspondence: Joana Costa: jcosta@uc.pt

Table S1. *L. pneumophila* CFU/ml corresponding to an OD600 of 1, assessed by the drop plate method.

**OD_{600}= 1**	
***L. pneumophila* strain**	**CFU/ml**
HRD2	3,8 x 10^9
IMC23	7,8 x 10^9
Lansing3 (ATCC 35251)	5,5 x 10^9
Los Angeles1 (ATCC 33156^T)	4,3 x 10^9
Philadelphia1 (ATCC 33152^T)	1,9 x 10^9
HUC1	5,8 x 10^9
MICU B (ATCC 33735)	2,0 x 10^9
Por3	2,6 x 10^8
U8W (ATCC 33737^T)	2,0 x 10^9
Ice27	3,2 x 10^9
Aço12	3,1 x 10^9
Aço22	3,5 x 10^9
Aço5	6,5 x 10^9
Agn2	6,1 x 10^9
Ger10	6,3 x 10^9
NMex1	3,9 x 10^9
