# Supplementary material for: Differences in Virulence Between Legionella pneumophila Isolates From Human and Non-human Sources Determined in Galleria mellonella Infection Model
Source: Front Cell Infect Microbiol. 2018 Apr 4;8:97. doi: 10.3389/fcimb.2018.00097 (PMC5893783; doi:10.3389/fcimb.2018.00097)
Supplement: Supplementary file 3 [file Table2.PDF]

Differences in virulence between *Legionella pneumophila* isolates from human and non-human sources determined in *Galleria mellonella* infection model

Patrícia Singéis de Sousa, Inês N. Silva, Leonilde M. Moreira, António Veríssimo, Joana Costa

Table S2. Survival and phenotypic characteristics after injection with 10^6 CFU per larvae of the tested *L. pneumophila* strains

***L. pneumophila* strain HRD2 (Clinical- related)**

Hours post injection	Survival		Melanization				Cocoon formation		Activity		
	Dead	Alive	No	Spots	Black spots	Black	Full	No	Move without stimulation	Move when stimulated	No movement
18	0	10	0	8	2	0	7	3	0	10	0
18	0	10	0	7	3	0	9	1	0	10	0
18	0	10	0	6	4	0	9	1	0	10	0
24	10	0	0	0	0	0	0	0	0	0	0
24	10	0	0	0	0	0	0	0	0	0	0
24	10	0	0	0	0	0	0	0	0	0	0
48											
48											
48											
72											
72											
72											

***L. pneumophila* strain Lansing3 (ATCC 35251) (Clinical- related)**

Hours post injection	Survival		Melanization				Cocoon formation		Activity		
	Dead	Alive	No	Spots	Black spots	Black	Full	No	Move without stimulation	Move when stimulated	No movement
18	4	6	1	5	0	0	5	1	0	3	3
18	4	6	6	0	0	0	6	0	6	0	0
18	4	6	1	4	1	0	4	2	4	0	2
24	5	5	2	2	1	0	1	4	0	0	5
24	4	6	3	3	0	0	2	4	6	0	0
24	4	6	2	3	1	0	2	4	0	4	2
48	6	4	0	3	1	0	2	2	0	1	3
48	5	5	3	2	0	0	3	2	0	5	0
48	6	4	1	3	0	0	4	0	0	0	4
72	6	4	0	2	2	0	1	3	0	0	4
72	5	5	0	2	3	0	2	3	0	0	5
72	6	4	0	1	3	0	4	0	0	0	4

***L. pneumophila* strain Los Angeles1 (ATCC 33156T) (Clinical- related)**

Hours post injection	Survival		Melanization				Cocoon formation		Activity		
	Dead	Alive	No	Spots	Black spots	Black	Full	No	Move without stimulation	Move when stimulated	No movement
18	2	8	4	4	0	0	3	5	0	5	3
18	2	8	7	1	0	0	3	5	8	0	0
18	1	9	1	7	0	1	7	2	8	0	1
24	2	8	4	2	2	0	4	4	0	8	0
24	2	8	6	2	0	0	3	5	0	8	0
24	2	8	7	1	0	0	4	4	0	8	0
48	2	8	0	7	1	0	4	4	0	6	2
48	2	8	0	7	0	1	5	3	5	2	1
48	2	8	0	7	0	1	4	4	4	3	1
72	4	6	0	4	0	2	4	2	0	0	6
72	3	7	0	7	0	0	5	2	0	0	7
72	4	6	0	6	0	0	3	3	0	0	6

***L. pneumophila* strain Philadelphia1 (ATCC 33152T) (Clinical- related)**

Hours post injection	Survival		Melanization				Cocoon formation		Activity		
	Dead	Alive	No	Spots	Black spots	Black	Full	No	Move without stimulation	Move when stimulated	No movement
18	0	10	9	1	0	0	10		10	0	0
18	0	10	7	3	0	0	8	2	8	2	0
18	0	10	8	2	0	0	9	1	8	2	0
24	0	10	6	4	0	0	5	5	4	6	0
24	0	10	6	3	1	0	5	5	3	7	0
24	0	10	6	4	0	0	4	6	2	8	0
48	2	8	2	6	0	0	3	8	2	6	0
48	4	6	0	5	1	0	1	5	0	4	2
48	4	6	0	6	0	0	0	6	0	5	1
72	4	6	0	0	6	0	0	6	0	0	6
72	5	5	0	1	4	0	0	5	0	1	4
72	6	4	0	1	3	0	0	4	0	0	4

***L. pneumophila* strain HUC1 (Man-made environment)**

Hours post injection	Survival		Melanization				Cocoon formation		Activity		
	Dead	Alive	No	Spots	Black spots	Black	Full	No	Move without stimulation	Move when stimulated	No movement
18	2	8	1	4	3	0	4	4	0	4	4
18	3	7	0	5	2	0	3	4	0	2	5
18	3	7	1	4	2	0	4	3	0	7	0
24	5	5	0	5	0	0	0	5	0	0	5
24	6	4	0	3	1	0	0	4	0	0	4
24	6	4	1	2	1	0	0	4	0	0	4
48	9	1	0	0	1	0	0	1	0	0	1
48	0	0	0	0	0	0	0	0	0	0	0
48	0	0	0	0	0	0	0	0	0	0	0
72	0	0	0	0	0	0	0	0	0	0	0
72	0	0	0	0	0	0	0	0	0	0	0
72	0	0	0	0	0	0	0	0	0	0	0

***L. pneumophila* strain IMC23 (Man-made environment)**

Hours post injection	Survival		Melanization				Cocoon formation		Activity		
	Dead	Alive	No	Spots	Black spots	Black	Full	No	Move without stimulation	Move when stimulated	No movement
18	0	10	1	9	0	0	7	3	2	8	0
18	0	10	10	0	0	0	10	0	7	3	0
18	0	10	5	5	0	0	8	2	8	2	0
24	0	10	8	2	0	0	6	4	4	6	0
24	0	10	6	4	1	0	4	6	3	7	0
24	0	10	3	6	1	0	4	6	4	5	1
48	2	8	0	4	2	2	0	8	0	7	1
48	1	9	0	6	2	1	2	7	0	5	4
48	2	8	0	3	4	1	0	8	0	5	3
72	8	2	0	0	1	1	0	2	0	0	2
72	6	4	0	0	2	2	0	4	0	0	4
72	8	2	0	0	1	1	0	2	0	0	2

***L. pneumophila* strain MICU B (ATCC 33735) (Man-made environment)**

Hours post injection	Survival		Melanization				Cocoon formation		Activity		
	Dead	Alive	No	Spots	Black spots	Black	Full	No	Move without stimulation	Move when stimulated	No movement
18	10	0	0	0	0	0	0	0	0	0	0
18	10	0	0	0	0	0	0	0	0	0	0
18	10	0	0	0	0	0	0	0	0	0	0
24											
24											
24											
48											
48											
48											
72											
72											
72											

***L. pneumophila* strain U8W (ATCC 33737T) (Man-made environment)**

Hours post injection	Survival		Melanization				Cocoon formation		Activity		
	Dead	Alive	No	Spots	Black spots	Black	Full	No	Move without stimulation	Move when stimulated	No movement
18	2	8	7	1	0	0	6	2	0	0	8
18	0	10	7	3	0	0	7	0	0	10	0
18	0	10	6	4	0	0	10	0	0	10	0
24	2	8	6	2	0	0	4	4	0	0	8
24	0	10	8	2	0	0	0	10	0	10	0
24	0	10	7	3	0	0	0	10	0	5	5
48	6	4	0	4	0	0	4	0	0	2	2
48	4	6	3	3	0	0	0	6	0	0	6
48	3	7	0	5	2	0	0	7	0	0	7
72	10	0	0	0	0	0	0	0	0	0	0
72	10	0	0	0	0	0	0	0	0	0	0
72	8	2	0	0	0	2	0	2	0	0	2

***L. pneumophila* strain Ice27 (Natural environment)**

Hours post injection	Survival		Melanization				Cocoon formation		Activity		
	Dead	Alive	No	Spots	Black spots	Black	Full	No	Move without stimulation	Move when stimulated	No movement
18	2	8	6	1	1	0	6	0	6	2	0
18	2	8	8	0	0	0	8	0	8	0	0
18	2	8	8	0	0	0	8	0	8	0	0
24	2	8	6	1	1	0	0	8	0	7	1
24	2	8	6	2	1	1	0	8	0	7	1
24	2	8	8	0	0	0	1	0	8	0	0
48	4	6	0	5	1	0	3	0	2	3	1
48	5	5	3	2	0	0	3	0	0	5	0
48	4	6	0	2	0	0	2	0	0	6	0
72	5	5	1	3	1	0	0	5	0	0	5
72	5	5	1	2	2	0	2	3	0	4	1
72	4	6	0	3	3	0	0	6	0	6	0

***L. pneumophila* strain Aço12 (Natural environment)**

Hours post injection	Survival		Melanization				Cocoon formation		Activity		
	Dead	Alive	No	Spots	Black spots	Black	Full	No	Move without stimulation	Move when stimulated	No movement
18	0	10	8	2	0	0	10	0	9	1	0
18	0	10	9	1	0	0	9	1	8	2	0
18	0	10	10	0	0	0	10	0	10	0	0
24	0	10	7	3	0	0	8	2	1	9	0
24	0	10	8	2	0	0	6	4	7	3	0
24	0	10	7	3	0	0	7	3	5	5	0
48	3	7	2	5	0	0	3	4	1	6	0
48	2	8	1	7	0	0	3	5	3	5	0
48	1	9	0	9	0	0	2	7	0	9	0
72	4	6	4	2	0	0	5	1	0	6	0
72	3	7	5	2	0	0	5	2	1	6	0
72	2	8	4	4	0	0	4	4	2	6	0

***L. pneumophila* strain Aço22 (Natural environment)**

Hours post injection	Survival		Melanization				Cocoon formation		Activity		
	Dead	Alive	No	Spots	Black spots	Black	Full	No	Move without stimulation	Move when stimulated	No movement
18	0	10	7	3	0	0	10	0	3	7	0
18	0	10	10	0	0	0	10	0	9	1	0
18	0	10	9	1	0	0	9	1	8	2	0
24	0	10	7	3	0	0	9	1	9	1	0
24	0	10	9	1	0	0	10	0	0	10	0
24	0	10	8	2	0	0	9	1	2	8	0
48	2	8	4	4	0	0	3	5	1	6	1
48	3	7	3	3	1	0	2	5	2	5	0
48	3	7	2	5	0	0	3	4	1	4	2
72	7	3	0	1	2	0	0	3	0	1	2
72	6	4	0	1	3	0	0	4	0	0	4
72	7	3	0	0	3	0	0	3	0	1	2

***L. pneumophila* strain Aço5 (Natural environment)**

Hours post injection	Survival		Melanization				Cocoon formation		Activity		
	Dead	Alive	No	Spots	Black spots	Black	Full	No	Move without stimulation	Move when stimulated	No movement
18	0	10	9	1	0	0	10	0	6	4	0
18	0	10	10	0	0	0	10	0	7	3	0
18	0	10	8	2	0	0	9	1	6	4	0
24	0	10	8	2	0	0	5	5	5	5	0
24	0	10	9	1	0	0	6	4	4	6	0
24	0	10	7	2	1	0	5	5	5	5	0
48	1	9	0	5	5	0	1	8	2	4	3
48	0	10	0	7	3	0	2	8	1	9	0
48	2	8	0	6	2	0	1	7	0	5	3
72	7	3	0	0	3	0	0	3	0	1	2
72	8	2	0	0	2	0	0	2	0	0	2
72	8	2	0	0	2	0	0	2	0	0	2

***L. pneumophila* strain Agn2 (Natural environment)**

Hours post injection	Survival		Melanization				Cocoon formation		Activity		
	Dead	Alive	No	Spots	Black spots	Black	Full	No	Move without stimulation	Move when stimulated	No movement
18	6	4	1	0	3	0	2	2	0	1	3
18	4	6	0	4	2	0	3	3	0	0	6
18	5	5	0	4	1	0	1	4	0	0	5
24	8	2	1	0	0	1	0	2	0	0	2
24	7	3	0	3	0	0	0	3	0	0	3
24	8	2	0	2	0	0	0	2	0	0	2
48	10	0	0	0	0	0	0	0	0	0	0
48	9	1	0	1	0	0	0	1	0	0	1
48	10	0	0	0	0	0	0	0	0	0	0
72											
72	10	0	0	0	0	0	0	0	0	0	0
72											

***L. pneumophila* strain Ger10 (Natural environment)**

Hours post injection	Survival		Melanization				Cocoon formation		Activity		
	Dead	Alive	No	Spots	Black spots	Black	Full	No	Move without stimulation	Move when stimulated	No movement
18	2	8	6	2	0	0	0	0	0	0	8
18	2	8	0	7	1	0	3	0	0	7	1
18	2	8	2	5	1	0	2	6	4	4	0
24	6	4	0	0	4	0	4	0	0	0	4
24	4	6	4	2	0	0	6	0	0	0	6
24	5	5	0	5	0	0	2	3	0	5	0
48	7	3	0	0	0	0	2	0	0	0	3
48	6	4	0	4	0	0	1	0	0	2	2
48	6	4	0	3	1	0	0	4	0	3	1
72	10	0	0	0	0	0	0	0	0	0	0
72	10	0	0	0	0	0	0	0	0	0	0
72	10	0	0	0	0	0	0	0	0	0	0

***L. pneumophila* strain NMex1 (Natural environment)**

Hours post injection	Survival		Melanization				Cocoon formation		Activity		
	Dead	Alive	No	Spots	Black spots	Black	Full	No	Move without stimulation	Move when stimulated	No movement
18	0	10	0	7	3	0	7	3	0	10	0
18	0	10	0	7	3	0	8	2	0	10	0
18	0	10	0	8	2	0	9	1	0	10	0
24	10	0	0	0	0	0	0	0	0	0	0
24	10	0	0	0	0	0	0	0	0	0	0
24	10	0	0	0	0	0	0	0	0	0	0
48											
48											
48											
72											
72											
72											
